# Increased ferritin, serum lactate dehydrogenase, and aspartate aminotransferase levels predict macrophage activation syndrome complicating systemic lupus erythematosus: a retrospective study

**DOI:** 10.3389/fped.2024.1469912

**Published:** 2024-12-17

**Authors:** Yingying Liu, Yuting Pan, Jing Jin, Panpan Wang, Tonghao Zhang, Zhidan Fan, Haiguo Yu

**Affiliations:** Department of Rheumatology and Immunology, Children’s Hospital of Nanjing Medical University, Nanjing, China

**Keywords:** ferritin, lactate dehydrogenase, aspartate aminotransferase, childhood-onset systemic lupus erythematosus, macrophage activation syndrome

## Abstract

**Background:**

This study aimed to assess the diagnosis of macrophage activation syndrome (MAS) at the onset of active childhood-onset systemic lupus erythematosus (cSLE), which is under-researched, and to compare the characteristics of cSLE with and without MAS, hypothesizing the existence of possible predictors of MAS in active cSLE.

**Methods:**

This study enrolled 157 patients diagnosed with cSLE, with or without MAS, from Nanjing Medical University between January 2018 and May 2023. Data analysis was performed using an independent samples *t*-test or the Mann–Whitney *U*-test, the *χ*^2^ test, the Youden index to determine the optimal cutoff values for diagnosis, and binary logistic regression analysis to determine the predicted probability.

**Results:**

Fifteen patients (9%) had MAS in the active phase, with an SLE disease activity index of 16.6 (range, 6–32). Bone marrow aspirations revealed hemophagocytosis in 8/15 cases (53%). Fever was the most common feature of MAS patients. Lactate dehydrogenase (LDH) and ferritin levels were elevated in the patients. Lower leukocyte, neutrophil, and platelet counts, including serum sodium and fibrinogen, and increased alanine aminotransferase, aspartate aminotransferase (AST), lactate dehydrogenase (LDH), ferritin, triglyceride, and D-dimer levels occurred in MAS patients, unlike those without MAS. Optimal cutoff values for ferritin (≥607.35 ng/ml), LDH (≥424 U/L), and AST (≥61 U/L) were predictors of MAS occurrence in cSLE. No MAS patients experienced recurrence during an 18-month mean follow-up.

**Conclusions:**

Despite the narrow scope of the study, elevated levels of ferritin, LDH, and AST may represent indicators of cSLE complicated by MAS. Early diagnosis and treatment may improve outcomes.

## 1 Introduction

Macrophage activation syndrome (MAS), also known as hemophagocytic syndrome in the context of a rheumatologic disorder (1), is a life-threatening complication of rheumatic diseases. MAS results from the excessive activation and expansion of T lymphocytes and macrophages, leading to proinflammatory cytokine hyperproduction and a hyperinflammatory condition (2), with mortality rates ranging from 8% to 22% in pediatric rheumatic diseases (3, 4). Clinically, MAS is characterized by persistent hyperthermia, decreased whole blood cell counts, hepatosplenomegaly, hepatic dysfunction, hyperferritinemia, and coagulation abnormalities (5). MAS is most common in children with systemic juvenile idiopathic arthritis (sJIA) (4, 6), although there has been a recent increase in cases among those with childhood-onset systemic lupus erythematosus (cSLE) (7–9). The preliminary diagnostic guidelines for MAS in cSLE, developed by Parodi et al. in 2009 (9), are frequently ignored because of self-imposed limitations.

SLE is a highly heterogeneous autoimmune disease with multiorgan involvement and multiple autoantibody abnormalities. Compared with adult-onset SLE (aSLE), cSLE may be more aggressive, with higher disease activity, greater organ involvement, and higher morbidity and mortality (10). Differentiating MAS from active cSLE can be challenging because of their shared features (11, 12). Atypical symptoms of Mas in children may impede accurate diagnosis, delaying treatment and worsening prognosis.

This retrospective study aimed to identify clinical and laboratory predictors for the early identification and diagnosis of MAS at the onset of active cSLE. We evaluated the demographics, clinical and laboratory data, treatment, and outcomes of 157 patients with cSLE, with or without MAS.

## 2 Materials and methods

### 2.1 Patients

This retrospective cohort study enrolled newly diagnosed SLE patients admitted to the Children's Hospital of Nanjing Medical University between January 2018 and May 2023. SLE diagnosis was based on the 2019 classification criteria by the European League Against Rheumatism and the American College of Rheumatology (13) in Supplementary Table S1.

### 2.2 MAS diagnosis

MAS was primarily diagnosed based on the opinions of pediatric rheumatologists, as described in the preliminary diagnostic guidelines for MAS proposed by Parodi et al. in 2009 (9). According to these diagnostic guidelines, patients were considered to have MAS if they met at least one clinical criterion, including fever, hepatomegaly, splenomegaly, hemorrhagic manifestations, or central nervous system dysfunction, and at least two laboratory criteria, such as ferritin level ≥ 500 µg/L, cytopenia involving two or more cell lineages, aminotransferase (AST) level > 40 units/L, triglyceride (TG) level > 178 mg/dl, fibrinogen level ≤ 1.50 g/L, and lactate dehydrogenase (LDH) ≥ 567 units/L. Additionally, we checked whether our patients met the 2016 sJIA with MAS classification criteria (14). The items of these different criteria sets are presented in Supplementary Table S2.

### 2.3 Data analysis

Demographic data, including age, sex, disease duration of SLE at MAS onset, and triggers of MAS onset, as well as clinical features, including fever, hepatomegaly, splenomegaly, lymphadenopathy, neuropsychiatric symptoms, kidney involvement, cardiovascular involvement, gastrointestinal symptoms, and pulmonary lesions, were recorded. Laboratory data, including leukocyte and platelet counts, erythrocyte sedimentation rate (ESR), hemoglobin, serum liver transaminase, LDH, ferritin, TG, sodium, albumin, C-reactive protein (CRP), complement components (C3 and C4), plasma fibrinogen, and D-dimer levels, were also collected. Lupus disease activity was evaluated using the SLE disease activity index (SLEDAI) (15). Bone marrow aspirations were examined for hemophagocytosis. Finally, the specific treatment regimens and MAS outcomes were evaluated. The requirement for informed consent was waived for this retrospective study.

### 2.4 Statistical analysis

Statistical analyses were performed using SPSS IBM Statistics software version 23.0 (IBM Corp., Armonk, NY, USA). Quantitative variables were expressed as median and range or mean ± standard deviation (SD); qualitative variables were expressed as numbers and percentages. Quantitative data were compared between the two groups using an independent samples *t*-test or the Mann–Whitney *U*-test. Categorical data were compared using the *χ*^2^ test. Statistical significance was considered at *P*-value < 0.05. The ability of laboratory data to differentiate MAS onset from active cSLE was evaluated using the area under the receiver operating characteristic (ROC) curve analysis. The Youden index was applied to determine the optimal cutoff values for diagnosis. A binary logistic regression analysis was used to determine the predicted probability.

## 3 Results

### 3.1 Demographic, clinical, and laboratory features of MAS

The features of patients with MAS are described in Table 1. Fifteen (eleven females and four males) of the 157 in-hospital SLE patients met the criteria for juvenile SLE-associated MAS. The average age at MAS onset was 11.6 years (SD, 1.5 years). The mean SLE duration at MAS onset was 6 days [interquartile range (IQR) 1–42 days]. The MAS classification criteria (2016) (14) were sensitive (0.73) and specific (0.99) for children with sJIA. All but two patients with cSLE fulfilled these criteria, as shown in Table 2.

**Table 1:** Clinical information and laboratory data of macrophage activation syndrome (MAS).

Features	Number of patients (%)
Gender (F, %)	11 (73)
Age at MAS diagnosis (mean ± SD, years)	11.6 ± 1.5
Median duration of jSLE at the onset of MAS (IQR, days)	6 (1, 42)
Fever	14 (93)
Hepatomegaly	5 (33)
Splenomegaly	4 (27)
Lymphadenopathy	12 (80)
Neurological involvement	4 (27)
Hemoglobin < 120 g/L	13 (87)
Platelets < 100 × 10^9^/L	8 (53)
Leukocytes < 4.0 × 10^9^/L	13 (87)
LDH > 250 U/L	15 (100)
AST > 40 U/L	13 (87)
ALT > 40 U/L	10 (67)
Ferritin > 500 ng/ml	15 (100)
Fibrinogen ≤ 1.5 g/L	7 (47)
Triglycerides ≥ 3 mmol/L	5 (33)
ESR > 20 mm/h	11 (73)
CRP > 8 mg/dl	4 (27)
Hemophagocytosis in the bone marrow	8 (53)

IQR, interquartile range; LDH, lactate dehydrogenase; AST, aspartate aminotransferase; ALT, alanine aminotransferase; ESR, erythrocyte sedimentation rate; CRP, C-reactive protein; F, female; jSLE, juvenile-onset systemic lupus erythematosus.

**Table 2:** Patients of macrophage activation syndrome (MAS) meeting the 2009 and 2016 classification criteria.

Patients	2009													Meeting 2016 classifi cation criteria	Meeting 2009 classifi cation criteria
	Hepatomegaly	Splenomegaly	Neuropsychiatric symptoms	WBC (×10^9^/L)	Hb (g/L)	LDH (U/L)	Hemophagocytose	Fever	PLT (×10^9^/L)	AST (U/L)	Ferritin (ng/ml)	TG (mmol/L)	Fibrino gen (g/L)		
								2016							
1	−	−	−	15.6	89	1,709	+	No	30	261	12,044	7.56	1.3	Yes	Yes
2	−	−	−	1.54	140	899	+	Yes	245	180	3,082	20.18	2.01	Yes	Yes
3	−	+	+	3.26	108	450	+	Yes	25	35	607.7	2.11	1.99	No	Yes
4	+	+	−	1.44	95	1,324	+	Yes	101	98	4,092	1.71	1.13	Yes	Yes
5	−	−	+	1.63	113	786	+	Yes	73	515	1,468	1.29	1.29	Yes	Yes
6	+	−	−	6.5	113	591	−	Yes	92	78	1,436	1.46	4.18	Yes	Yes
7	−	+	−	1.41	99	1,277	+	Yes	137	254	1,987.5	3.54	2.09	Yes	Yes
8	−	−	+	2	139	1,269	+	Yes	126	62	2,724	0.74	1.74	Yes	Yes
9	−	−	−	3.41	73	1,613	+	Yes	129	114	22,660	4.36	2.98	Yes	Yes
10	−	−	−	3.07	111	478	−	Yes	97	115	3,714	1.97	4.17	Yes	Yes
11	+	−	−	1.82	93	429	−	Yes	56	205	1,550	1.43	1.64	Yes	Yes
12	−	−	+	2.81	68	623	−	Yes	178	43	1,248.6	2.24	1.39	Yes	Yes
13	−	+	−	2.41	103	956	−	Yes	77	673	643.9	2.05	2.13	No	Yes
14	+	−	−	0.88	66	589	−	Yes	7	37	1,499.3	1.94	1.39	Yes	Yes
15	+	−	−	1.37	108	587	−	Yes	133	1,259	2,050	3.62	1.23	Yes	Yes

In 8/15 (53%) patients, the SLE diagnosis was concomitant with the first MAS episode. All patients with MAS were in the active phase, and their SLEDAI scores ranged from 6 to 32 (mean, 16.6). Four patients had one or more associated autoimmune disorders at MAS onset, including antiphospholipid syndrome (one patient), secondary hypothyroidism (two patients), secondary Sjögren syndrome (one patient), and acute autoimmune pancreatitis (one patient). Persistent fever was the main clinical manifestation (14/15; 93%), and lymphadenopathy was the second most common (12/15; 80%).

Patients with MAS had significantly elevated LDH and ferritin levels compared to patients without MAS. Over 85% (13/15) of the children with MAS had elevated serum AST levels. Reduced hemoglobin and leukocyte levels were observed in 13/15 patients (87%). Only 53% (8/15) of patients who underwent bone marrow examination exhibited hemophagocytosis.

### 3.2 MAS trigger factors, treatment, and outcomes

All patients developed MAS during active underlying SLE. One-third (5/15; 33%) of the patients diagnosed with MAS had concomitant evidence of pathogenic infection [two cases of Epstein–Barr virus (EBV), one *Mycoplasma pneumoniae*, two adenovirus, one herpes virus, one influenza B virus, and one deep fungal infection]. Certain causative pathogens among the infected patients included Epstein–Barr virus and adenovirus. The remaining patients were diagnosed with MAS without signs of infection or other triggers during the active phase of underlying systemic autoimmune diseases. No cases of drug-induced MAS have been reported.

All patients received corticosteroid therapy, and most patients (73%) also received high-dose methylprednisolone pulse therapy, as shown in Table 3. Some patients (53%) received intravenous (IV) high-dose methylprednisolone therapy (15–30 mg/kg/day) combined with low-dose IV methylprednisolone or oral prednisone (1–2 mg/kg/day) and CsA (4–6 mg/kg/day). Two patients (patients 11 and 13) recovered without additional immunosuppressive therapy, and they achieved remission after receiving a lower dose of IV methylprednisolone and oral prednisone. Patient 8 experienced headache, vomiting, abdominal discomfort, convulsions, and persistent epilepsy in the evening, 3 days after the first high-dose methylprednisolone pulse combined with CsA therapy. Subsequently, owing to uncontrolled disease, the patient underwent a second pulse therapy with additional intravenous immunoglobulin (IVIG). Patient 15 received methylprednisolone pulse therapy during which abdominal pain occurred. Based on the results of an abdominal CT and B ultrasound, this was considered acute autoimmune pancreatitis. Methylprednisolone was reduced in dosage and pulse therapy for 2 days, with the addition of immunoglobulin and cyclosporine. Considering the ineffective control of amylase and other related indicators during re-examination, CsA was discontinued, and cyclophosphamide (CTX) pulse therapy was administered, with a significant improvement.

**Table 3:** Triggers, treatment, and outcome of macrophage activation syndrome (MAS).

Patient	Triggers	Therapies, effectiveness	Follow-up (months)	Outcome
1	Autoimmune	IVIG pulse + CsA, effective	20	Well
2	Autoimmune	mPSL + IVIG pulse + CsA, effective	/	Well
3	Autoimmune	mPSL pulse + rituximab, effective	36	Well
4	Autoimmune	mPSL pulse + CsA, effective	32	Well
5	Autoimmune	mPSL pulse + CsA + CTX pulse, effective	30	Well
6	Infection	mPSL pulse, effective	15	Well
7	Autoimmune	mPSL + IVIG pulse + CsA, effective	30	Well
8	Autoimmune	first mPSL pulse + CsA + PSL, ineffective; second, mPSL pulse + CsA + IVIG, effective	12	Well
9	Autoimmune	mPSL pulse + CsA, effective	48	Well
10	Infection	mPSL pulse + CsA, effective	8	Well
11	Autoimmune	PSL, effective	7	Well
12	Autoimmune	mPSL + IVIG, effective	5	Well
13	Autoimmune	PSL, effective	4	Well
14	Autoimmune	PSL + CsA, effective	2	Well
15	Autoimmune	mPSL + IVIG pulse + CsA + CTX pulse, effective	1	Well

IVIG, intravenous immunoglobulin; mPSL, pulse methylprednisolone pulse therapy; CsA, cyclosporine A; CTX, cyclophosphamide.

None of the patients died during hospitalization. The mean follow-up time was 18 months (range, 1–48 months). Patient 2 was lost to follow-up. None of the patients with MAS with available data experienced a relapse during outpatient or inpatient follow-ups.

### 3.3 Comparison of clinical and laboratory data between MAS and non-MAS cohorts

Fever, lymphadenopathy, hepatomegaly, and pulmonary involvement were more common and more significant in patients with MAS compared with non-MAS cSLE, as is shown in Table 4. Compared with patients with cSLE without MAS, those with MAS onset had lower white blood cell (WBC), neutrophil, and platelet counts, lower serum sodium and fibrinogen levels, and increased alanine transaminase (ALT), AST, LDH, ferritin, TG, and D-dimer levels. In addition, patients with MAS were more likely to have splenomegaly, hemorrhagic manifestations, and neurological involvement, and less likely to have arthritis; however, these differences were not significant.

**Table 4:** Comparison of clinical and laboratory data between active childhood-onset systemic lupus erythematosus (cSLE) with and without macrophage activation syndrome (MAS).

Feature	SLE	MAS (*n* = 15)	Non-MAS (*n* = 142)	*P*
Clinical data				
Fever	97 (62)	14 (93)	83 (58)	0.008
Lymphadenopathy	81 (52)	12 (80)	69 (49)	0.021
Hepatomegaly	21 (13)	5 (33)	16 (10)	0.017
Splenomegaly	18 (11)	4 (27)	14 (10)	0.052
Kidney involvement	75 (48)	9 (60)	66 (46)	0.319
Neurological involvement	25 (16)	4 (27)	21 (15)	0.232
Hemorrhage	33 (21)	4 (27)	29 (20)	0.572
Arthritis	44 (28)	3 (20)	41 (29)	0.467
Nasopharyngeal ulceration	52 (33)	6 (40)	46 (33)	0.552
Pulmonary involvement	21 (13)	5 (33)	16 (11)	0.017
Laboratory data				
WBC (×10^9^/L)	4.08 (3.07–6.49)	2 (1.44–3.26)	4.27 (3.3–6.6)	<0.001
Neutrophil count (×10^9^/L)	2.06 (1.43–3.57)	1.42 (0.66–2.11)	2.18 (1.55–3.83)	0.004
Hb (g/L)	105.5 (93–118.75)	103 (89–113)	106 (93–119)	0.453
PLT (×10^9^/L)	152.5 (93.75–218.5)	97 (56–133)	163 (110–223)	0.005
ALT (U/L)	27 (15–54.75)	95 (31–270)	24 (14–46.5)	<0.001
AST (U/L)	36 (23–56.75)	115 (62–261)	33 (22–50)	<0.001
LDH (U/L)	319.5 (241–449.5)	786 (587–1,277)	296 (235–395)	<0.001
Ferritin (ng/ml)	238.1 (127–498.22)	1,987.5 (1,436–3,714)	256.9 (116.5–431.75)	<0.001
CRP (mg/L)	8 (1.84–8)	8 (0.53–18.48)	8 (1.94–8)	0.549
ESR (mm/h)	38 (21.25–66.75)	40 (14–76)	38 (21.5–65)	0.921
TG (mmol/L)	1.5 (0.99–2.32)	2.11 (1.64–4.17)	1.46 (0.97–2.21)	0.002
Serum sodium (mmol/L)	138.45 (136–140)	136 (124.1–139)	138.8 (136.1–140.25)	0.007
Albumin (g/L)	38.1 (34.5–42.15)	34 (32.6–39.2)	38.1 (34.85–42.45)	0.076
fibrinogen (g/L)	2.64 (2.17–3.12)	1.99 (1.3–2.98)	2.67 (2.27–3.13)	0.010
D-Dimer (ng/ml)	405.5 (203.25–745.75)	1,163 (435–6,263)	378 (184–706)	<0.001
C3 (g/L)	0.43 (0.27–0.66)	0.47 (0.24–0.67)	0.43 (0.30–0.66)	0.575
C4 (g/L)	0.07 (0.07–0.11)	0.08 (0.07–0.16)	0.07 (0.07–0.11)	0.148
SLEDAI	14 (8–20)	18 (9–21)	14 (8–20)	0.247

WBC, white blood cell count; Hb, hemoglobin; PLT, platelets; LDH, lactate dehydrogenase; AST, aspartate aminotransferase; ALT, alanine aminotransferase; ESR, erythrocyte sedimentation rate; CRP, C-reactive protein; TG, triglyceride; C3 and C4, complement components; SLEDAI, the SLE Disease Activity Index.

Categorical data were compared using the *χ*^2^ test. Quantitative data were compared using an independent samples Mann–Whitney *U*-test.

**Figure 1:**
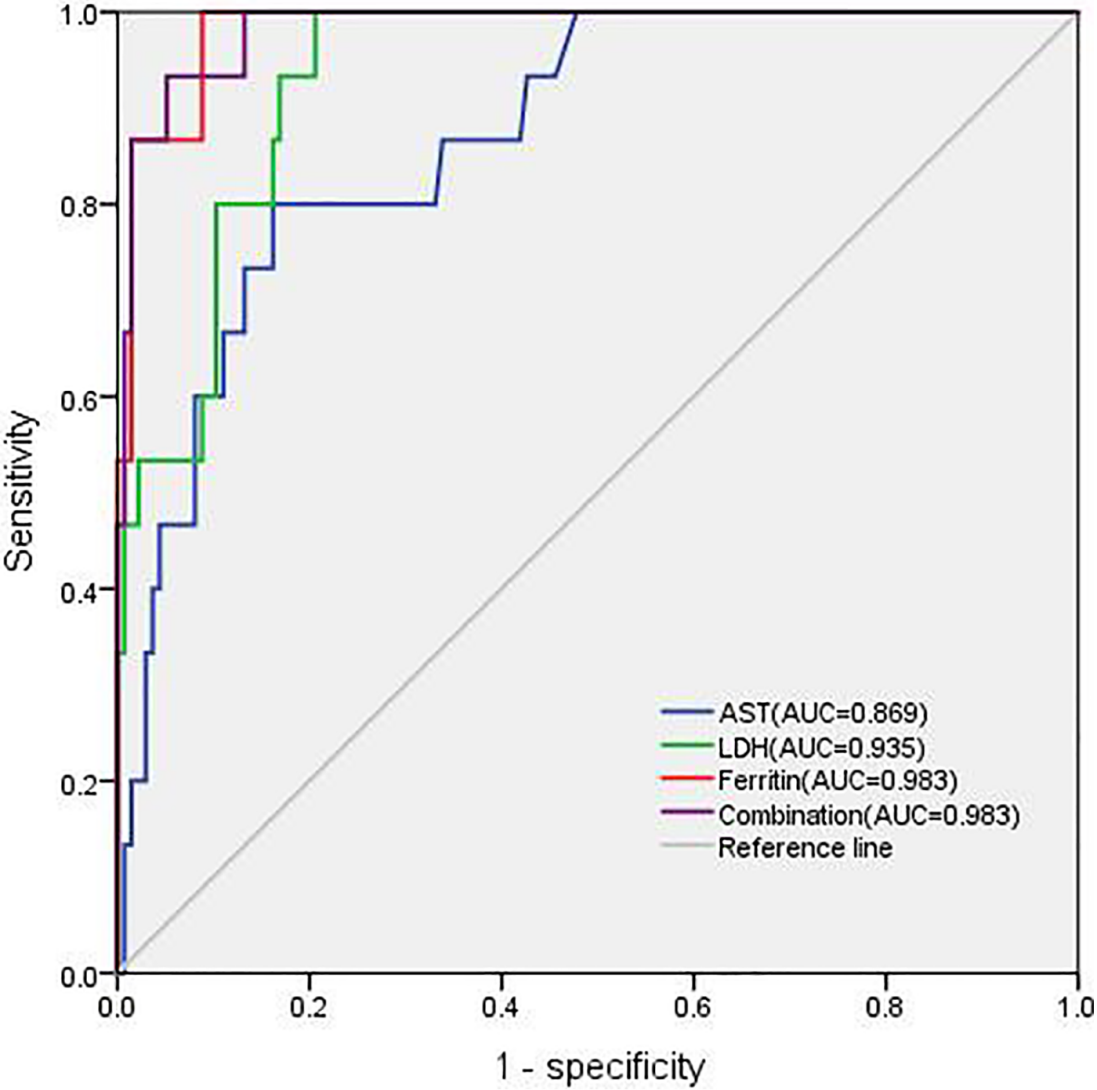
Different areas under the receiver operating characteristics curve (AUC) for AST, LDH, ferritin, and combination.

ROC curve analysis identified LDH and ferritin levels as the best predictors of MAS diagnosis. More specifically, cutoff values of ≥607.35 ng/ml for ferritin and ≥424 U/L for LDH can improve the early diagnosis of MAS in cSLE, as is shown in Table 5. In addition, AST had an 80% sensitivity and 84.5% specificity, with a high AUC (>0.8). Combining these three variables for the ROC analysis, the AUC reached 0.983 which was statistically significant (Figure 1).

**Table 5:** The result of ROC curve analysis for laboratory features between active childhood-onset systemic lupus erythematosus (cSLE) with and without macrophage activation syndrome (MAS).

Feature	ROC-AUC	cutoff value	Sensitivity (%)	Specificity (%)	95% CI	*P*
Leukocytes (×10^9^/L)	0.815	≤3.42	86.7	73.9	0.670–0.960	<0.0001
Platelets (×10^9^/L)	0.720	≤139.50	86.7	61.3	0.605–0.835	0.005
ALT (U/L)	0.798	≥24.50	100	49.3	0.696–0.900	<0.0001
AST (U/L)	0.874	≥61.00	80	84.5	0.794–0.954	<0.0001
LDH (U/L)	0.937	≥424.00	100	79.6	0.893–0.980	<0.0001
Ferritin (ng/ml)	0.983	≥607.35	100	91.2	0.965–1.000	<0.0001
TG (mmol/L)	0.742	≥1.63	80	62	0.607–0.877	0.002
Serum sodium (mmol/L)	0.712	≤137.05	73.3	64.8	0.557–0.867	0.007
Fibrinogen (g/L)	0.704	≤2.14	73.3	84.9	0.509–0.899	0.010
D-Dimer (ng/ml)	0.788	≥1,135.50	53.3	89.9	0.669–0.907	<0.0001

ALT, alanine aminotransferase; AST, aspartate aminotransferase; LDH, lactate dehydrogenase; TG, triglyceride.

## 4 Discussion

MAS is an underdiagnosed complication of cSLE because it is indistinguishable from some severe infections and SLE flare-ups. This study examined the clinical and laboratory features of patients with cSLE at the onset to facilitate early diagnosis and intervention. The prevalence of MAS in our cSLE cohort was 9%, similar to the 9% reported by Borgia et al. (7) and 7% reported by Aytaç et al. (16). Moreover, MAS was most likely to develop in conjunction with, or within 1 month of, SLE diagnosis. This is consistent with other cSLE-associated MAS reports (7, 17).

Although the etiology of MAS remains unclear, it is associated with a massive release of proinflammatory cytokines due to the excessive activation and proliferation of T lymphocytes as well as macrophages. Recent studies have reported that overproduction of type I IFN is involved in MAS development in aSLE (18). Usami et al. found that serum CXCL9 and sTNFR-II levels in SLE patients were significantly elevated during the MAS phase (19). Active disease is the main trigger factor for MAS, followed by infection, and certain medications. Here, all patients were diagnosed with MAS in the setting of active underlying SLE with a mean SLEDAI level of 16.6 (range, 6–32). An uncontrolled and high-activity SLE state may account for the increased risk of developing MAS. Pathogenic infection can be identified in some patients, and Epstein–Barr virus infection represented the most significant potential trigger for MAS. Furthermore, cytomegalovirus infection closely mimicked MAS; however, no cytomegalovirus infection was detected. Hence, patients suspected to have MAS should be screened for other infections.

Overlapping clinical and laboratory data, such as fever, organomegaly, and cytopenia, make it difficult to differentiate MAS. Fever was the most common clinical feature of patients with MAS in our cohort, as described in previous cSLE and aSLE cohorts (7, 8, 20), followed by lymphadenopathy. However, fever may not be an absolute indicator for every MAS case, as a patient in our cohort with ongoing immunosuppression and herpes virus infection did not present with fever at MAS onset. The absence of fever has also been reported in children treated with biologicals (21). The percentage of liver enlargement was significantly higher in patients with cSLE and MAS. Liver dysfunction helps in the further diagnosis of underlying MAS in patients with SLE.

Laboratory features are more sensitive than clinical manifestations for capturing the occurrence of MAS. Although cytopenia is also commonly seen in lupus, it may be a key to early diagnosis of MAS. Our patients showed a significant decrease in leukocytes and platelets, especially leukocytes, at the time of MAS onset. This result is difficult to understand in the context of underlying SLE, and MAS must be assessed. Hyperferritinemia is the strongest indicator for differentiating MAS from active SLE, with a sensitivity and specificity of almost 100% (22). Bennett et al. (23) concluded that high serum ferritin levels correlate with underlying disease severity; however, their relevance to disease activity remains disputable. In our cohort, laboratory variables were compared between cSLE patients with and without MAS onset. Obviously, ferritin and LDH were not high during active SLE. Ferritin and LDH were the strongest indicators for differentiating MAS with the highest AUC in the ROC analysis. Patients 3 and 13 did not meet the 2016 classification criteria (14) owing to the hyperferritinemia threshold (≥684 ng/ml); however, in our study, hyperferritinemia using the threshold of ≥607.35 ng/ml had the best sensitivity (100%) and specificity (91.2%). It is possible that different active disease backgrounds may lead to this variation. In addition, AST showed good discriminatory capacity sensitivity. Therefore, ferritin, LDH, and AST levels were the best predictors for distinguishing MAS from active cSLE, similar to previous studies (7, 9).

MAS is characterized by hemophagocytosis resulting from macrophage activation; increased hemophagocytic activity can be histopathologically demonstrated in the bone marrow, liver, and spleen (24). However, in our study, nearly half of SLE patients (47%) were diagnosed with MAS without apparent hemophagocytosis. Although hemophagocytosis may not be present in the initial stages and is not specific to MAS, bone marrow aspirates can help exclude other disease conditions, such as infections or malignancy. Hemophagocytosis in the bone marrow may be absent at disease onset, in which case MAS can be diagnosed based on typical clinical and laboratory evidence (9).

Timely intervention is important if patients are suspected of having a MAS infection. Currently, a standard treatment for this syndrome in rheumatologic diseases has yet to be established, and its management is based on experience generated from case series (25, 26). Intravenous pulse therapy with methylprednisolone (IVMP; 30 mg/kg/day, maximum 1 g/day for 3–5 days), followed by oral prednisolone, is the most commonly used first-line treatment (27). In our study, all patients achieved remission after receiving corticosteroids alone or combined with IVIG and immunosuppressant therapy. Over 70% of the patients with persistent high fever achieved remission with high-dose methylprednisolone pulse therapy. If adequate glucocorticoids are ineffective in SLE patients with persistently high fever, MAS may be a factor. If patients have SLE with MAS, especially with multisystem organ damage, pulsed methylprednisolone may be the preferred initial immunosuppressive agent (28).

The choice of immunosuppressants for SLE patients with MAS, with most rheumatologists preferring CsA to cyclophosphamide (CTX) due to bone marrow suppression induced by intravenous CTX, is controversial. CsA has been considered as a therapeutic option for refractory lupus nephritis with persistent severe proteinuria and hematological involvement (29). CsA, a calcineurin inhibitor, has been proven to be life-saving in steroid-resistant and refractory MAS (30), especially in MAS associated with sJIA. Up to 60% (9/15) of the patients in our cohort received additional CsA, with a prognosis.

IVIG is another therapeutic option for MAS that can be used as an adjuvant treatment to IVMP (31). Although IVIG can be the initial therapy if the clinically concomitant infection is clear, other immunosuppressive treatments, such as CTX and etoposide, are recommended as ineffective first-line treatments. Successful outcomes can be obtained with CTX in patients with MAS refractory to initial IVMP (32). As in our study, patient 5 first received IVMP pulse therapy (1,000 mg/day) for 3 days, followed by low-dose IV prednisolone (2 mg kg) and CsA treatment. After 10 days of MAS, the patient presented with kidney involvement and massive proteinuria. Therefore, CsA treatment was discontinued, and CTX shock therapy was initiated, with eventual clinical remission. Therefore, CTX should be a priority for patients presenting with refractory nephritis, especially proliferative nephritis, but it should be used with caution in patients with underlying immunosuppression due to bone marrow suppression.

Biological agents targeting cytokines are increasingly used for the treatment of MAS. Anakinra is used for MAS in the setting of an underlying rheumatic disease, especially sJIA unresponsive to corticosteroids and CsA, with remarkable efficacy (33, 34). However, the benefits of treating MAS secondary to SLE remain unclear and require further clinical trials. Other anti-cytokine therapeutic options, such as TNF, IL-6, and IL-18 blockers, have been applied to MAS, and IFN-*γ* is emerging as a new therapeutic target (25). In our study, rituximab, a monoclonal antibody against CD20, was used in one case of MAS with uncontrolled disease. Rituximab has been employed in MAS triggered by the Epstein–Barr virus with promising outcomes in a retrospective investigation (35).

None of our patients died during treatment, possibly due to early identification and prompt treatment, and none experienced a recurrence of MAS during a mean follow-up of 2 years, which agrees with previously reported studies (7, 17). Although MAS recurrence may be more frequently observed in sJIA and aSLE (36, 37), few cases have been reported in cSLE (11, 12, 22).

The limitations of this study include the retrospective, single-center, small-sample format and lack of cellular or molecular evaluation (sCD25, sCD163, and genetic assessment). Additionally, the short-term overall prognosis was good; however, we did not track and compare the long-term laboratory results and therapeutics of patients with SLE, with or without MAS. The validated MAS criteria in SLE are not available, using the Parodi criteria may lead to an inclusion bias in this study. The validation cohort cannot be conducted due to the limited number of cases. More cases will be acquired in the future to validate our results. More international multicenter studies on MAS in cSLE may further evaluate the risk factors, therapeutic strategies, and long-term prognoses.

## 5 Conclusions

In conclusion, our findings suggest that clinical and laboratory features aid in diagnosing MAS early in patients with active cSLE, and patients with active cSLE should be considered to have MAS if they present with persistent fever and elevated ferritin, LDH, and AST levels. These findings may contribute to the timely therapeutic management of MAS.

## Data Availability

The original contributions presented in the study are included in the article/Supplementary Material, further inquiries can be directed to the corresponding authors.
