# Supplementary material for: Increased ferritin, serum lactate dehydrogenase, and aspartate aminotransferase levels predict macrophage activation syndrome complicating systemic lupus erythematosus: a retrospective study
Source: Front Pediatr. 2024 Dec 17;12:1469912. doi: 10.3389/fped.2024.1469912 (PMC11685012; doi:10.3389/fped.2024.1469912)
Supplement: Supplementary file 1 [file Table1.docx]

**Supplementary Table 1.** 2019 EULAR/ACR classification criteria in systemic lupus erythematosus.

| **Clinical area or criterion** | **Definition** | **Weighting** |
| --- | --- | --- |
| Systemic conditions | Fever | 2 |
| Skin mucosa | Non-scarring alopecia | 2 |
|  | Oral ulcers | 2 |
|  | Subacute cutaneous lupus or discoid lupus | 4 |
|  | Acute cutaneous lupus | 6 |
| Joints | Synovitis/swelling/fluid in >2 joints, mobility in >2 joints  Limitations and morning stiffness >30min | 6 |
| Nervous system | Delirium | 2 |
|  | Mental abnormality | 3 |
|  | Epilepsy | 5 |
| Plasmacytitis | Pleural or pericardial effusion | 5 |
|  | Acute pericarditis | 6 |
| Hematologic system | Leukopenia <4×10^9／L | 3 |
|  | Thrombocytopenia <100×10^9／L | 4 |
|  | Hemolytic anemia | 4 |
| Kidney | Proteinuria ＞0.5g/24h | 4 |
|  | Renal biopsy: type II or V LN | 8 |
|  | Renal biopsy: type III or type IV LN | 10 |
| Antiphospholipid antibodies | Positive anticardiolipin antibodies or anti-beta2 glycoprotein or lupus anticoagulant | 2 |
| Complement | Low C3 or low C4 | 3 |
|  | Low C3 and low C4 | 4 |
| Specific Antibody | Positive anti-dsDNA antibody | 6 |
|  | Positive anti-Smith antibody | 6 |

*EULAR/ ACR:European League Against Rheumatism/American College of Rheumatology ; LN.lupus nephritis;dsDNA.double - stranded DNA; Entry criterion: antinuclear antibody at a titre of ≥1:80 on HEp -2 cells or an equivalent positive test. Additive eriteria, De noi count a criterion if there is a more likely explanation than systemie lupus erthematosus. Occurrence of a eriterion on at least one clinical eriterioe and ≥10 points. Criteria need not occur simultancously. Within each domain, only the highest weighted criterion is counted toward the total scores.*

**Supplementary Table 2.** The diagnostic criteria for macrophage activation syndrome (MAS) by Ravelli et al for systemic juvenile arthritis (SJIA)-associated MAS

| **Criteria by Ravelli et al for SJIA-associated MAS (reference number 14)** |
| --- |
| -Diagnosis of MAS: A febrile patients with known or suspected SJIA and ferritin > 684 ng/mL AND ≥2 of 4 criteria below |
| 1. Platelet <181,000/mm^3^ |
| 1. AST > 48 U/L |
| 1. TG > 156 mg/dL |
| 1. Fibrinogen ≤ 360 mg/dL |

*AST, aspartate aminotransferase; LDH, lactate dehydrogenase; MAS, macrophage activation syndrome; SJIA, systemic juvenile idiopathic arthritis; SLE, systemic lupus erythematosus; TG, triglyceride; WBC, white blood cell*
